# Hypophyseal Involvement in Immunoglobulin G4-Related Disease: A Retrospective Study from a Single Tertiary Center

**DOI:** 10.1155/2018/7637435

**Published:** 2018-03-20

**Authors:** Yang Liu, Linjie Wang, Wen Zhang, Hui Pan, Hongbo Yang, Kan Deng, Lin Lu, Yong Yao, Shi Chen, Xiaofeng Chai, Feng Feng, Hui You, Zimeng Jin, Huijuan Zhu

**Affiliations:** ^1^Department of Neurosurgery, Peking Union Medical College, Beijing 100730, China; ^2^Key Laboratory of Endocrinology of National Health and Family Planning Commission, Department of Endocrinology, Peking Union Medical College, Beijing 100730, China; ^3^Department of Rheumatology, Peking Union Medical College, Beijing 100730, China; ^4^Department of Radiology, Peking Union Medical College Hospital, Chinese Academy of Medical Science and Peking Union Medical College, Beijing 100730, China

## Abstract

This study aims to outline the clinical features and outcomes of IgG4-related hypophysitis (IgG4-RH) patients in a tertiary medical center. We reviewed clinical manifestations and imaging and pituitary function tests at baseline, as well as during follow-up. Ten patients were included. The mean age at diagnosis of IgG4-RH was 48.4 (16.0–64.0) years. An average of 3 (0–9) extrapituitary organs were involved. Five patients had panhypopituitarism, three had only posterior hypopituitarism, one had only anterior hypopituitarism, and one had a normal pituitary function. One patient in our study had pituitary mass biopsy, lacking IgG4-positive cells despite lymphocyte infiltration forming an inflammatory pseudotumor. Five patients with a clinical course of IgG4-RH less than nine months and a whole course of IgG4-RD less than two years were managed with glucocorticoids, while three patients with a longer history were administered glucocorticoids plus immunosuppressive agents. One patient went through surgical excision, and one patient was lost to follow-up. All patients showed a prompt response clinically, but only three patients had normalized serum IgG4 levels. Two patients who took medications for less than six months relapsed. *Conclusions*. IgG4-RD is a broad disease, and all physicians involved have to be aware of the possibility of pituitary dysfunction. Younger patients should be expected. The histopathological feature of pituitary gland biopsy could be atypical. For patients with a longer history, the combination of GC and immunosuppressive agents is favorable. Early and adequate courses of treatment are crucial for the management of IgG4-RH. With GC and/or immunosuppressant treatment, however, pituitary function or diabetes insipidus did not improve considerably.

## 1. Introduction

Immunoglobulin G4-related disease (IgG4-RD) [1] is a chronic systemic disease that involves multiple organs synchronously or metachronously and is characterized by the tissue infiltrated by lymphocytes, IgG4-positive plasma cells, fibrosis, and often, but not always, elevated serum IgG4 levels. Common manifestations include type I autoimmune pancreatitis (AIP), swelling of salivary and lacrimal glands (Mikulicz's disease), retroperitoneal fibrosis, and lymphadenopathy. The involvement of other systems, such as the lung, liver, kidney, and central nervous system, is not rare in the literature. Recently, hypophyseal involvement in IgG4-RD has been reported and has been added to the IgG4-RD spectrum, and it has also been well recognized as IgG4-related hypophysitis (IgG4-RH). Typically, pituitary masses in IgG4-RH patients demonstrate a dense lymphoplasmacytic infiltrate among residual nests of adenohypophyseal cells and fibrosis in histopathological analysis. On histology, IgG4-RH is essentially similar to lymphocytic hypophysitis, which is the most prevalent type of primary hypophysitis, besides massive IgG4-positive plasmacyte infiltration [2]. Lymphocytic hypophysitis shows lymphocytic infiltrations of varying density, predominantly of the T cell type [3]. The presence of IgG4-related systemic disease and an elevated serum IgG4 level were the main clues to a correct diagnosis of IgG4-RH. Autoimmunity is suggested but not established to play a role in the pathogenesis for IgG4-related systemic disease [4]. This disorder has been classified as a new form of primary hypophysitis associated with IgG4-related systemic disease [5].

Since 2011, when Leporati proposed new diagnostic criteria for the disease without the need for pituitary biopsy [6], IgG4-RH has been recognized by physicians and neurosurgeons. As one of several types of hypophysitis, IgG4-RH shares common clinical manifestations during the inflammatory process of the pituitary gland. Associated symptoms include headache, visual defects, hypopituitarism, and central diabetes insipidus (CDI) [7]. It is believed to likely develop in elderly or middle-aged men, and it responds promptly to glucocorticoid (GC) administration.

There have been several case reports and reviews regarding IgG4-RH in the literature. However, because of its low prevalence, a very limited number of clinical studies from a single medical center were published. In this study, we outlined the clinical, radiological, and pathological features of ten patients diagnosed with IgG4-RH in a tertiary medical center and pointed features different from previous reports. We also focused on the treatment and outcome of our patients and proposed a recommended treatment protocol.

## 2. Patients and Methods

### 2.1. Patient Information

All of the studies were performed according to the rules of the hospital's medical ethics committee. Informed consent was obtained in accordance with the institutional guidelines.

Data from all of the patients diagnosed with IgG4-RD in our center were reviewed, and those with pituitary involvement were noted. Clinical data from 10 patients diagnosed with IgG4-related hypophysitis at the Peking Union Medical College Hospital from December 2012 to January 2017 were retrospectively analyzed. Parameters collected include clinical manifestations and imaging and pituitary function tests at baseline and during follow-up.

### 2.2. Diagnosis of IgG4-RH

The diagnosis of IgG4-RH was made according to Leporati's diagnostic criteria [6]: (1) the presence of a mononuclear cell infiltration of the pituitary gland that is rich in lymphocytes and plasma cells, with more than ten IgG4-positive cells per high-power field or IgG4-positive/IgG-positive cells > 40% on pituitary histopathology, (2) the presence of a sellar mass and/or thickened pituitary stalk on MRI, (3) a biopsy proving the involvement in other organs (association with IgG4-positive lesions in other organs), (4) an increased serum IgG4 level (>140 mg/dl), and (5) a prompt shrinkage of the pituitary mass and symptom improvement with steroids. Diagnosis was made when criteria (1), or (2) and (3), or (1), (4), and (5) were fulfilled.

### 2.3. Evaluation of Pituitary Function

All patients went through endocrine examination regarding pituitary function, including measurements of adrenocorticotropic hormone (ACTH), cortisol, thyroid-stimulating hormone (TSH), free tetraiodothyronine (fT4), growth hormone (GH), insulin-like growth factor 1 (IGF1), FSH/LH, testosterone (T), estradiol (E2), urinary volume, urine, and plasma osmolality. Anterior pituitary hypofunction was defined as follows: GH deficiency was confirmed when low age-adjusted IGF1 levels were present with additional three pituitary deficiencies. Secondary adrenal deficiency was diagnosed if a symptomatic patient demonstrated low morning serum cortisol (<5 *μ*g/dl) and ACTH levels (<15 pg/ml). Secondary hypogonadism was defined as FSH/LH concentration that was not elevated for women with amenorrhoea/oligomenorrhea or for adult men with low serum testosterone levels; secondary hypothyroidism was diagnosed when serum fT4 levels were below the normal range and serum TSH level was inappropriately low or normal. The diagnosis of CDI was based on the clinical findings of polyuria and polydipsia, low urine osmolality (<300 mOsm/kg H_2_O) in a water deprivation test, and an increase in urinary osmolality or a decrease in urine volumes in response to a desmopressin trial.

### 2.4. Statistical Analysis

Data were statistically analyzed using GraphPad Prism, version 6 (GraphPad Software Inc., La Jolla, CA, USA). Descriptive data are shown as mean ± SD.

## 3. Results

Until January 2017, 262 patients had been diagnosed with IgG4-RD at the Peking Union Medical College Hospital, 10 (3.8%) of whom had pituitary involvement and were diagnosed with IgG4-RH. The mean age at diagnosis of IgG4-RH was 48.4 (16.0–64.0) years, with a male to female ratio of 4 : 1. The patients made their first visits in the departments of endocrinology (3 cases), rheumatology (2 cases), neurosurgery (2 cases), nephrology (1 case), general internal medicine (1 case), and stomatology (1 case).

### 3.1. Clinical Characteristics of IgG4-Related Hypophysitis Patients

**Figure 1:**
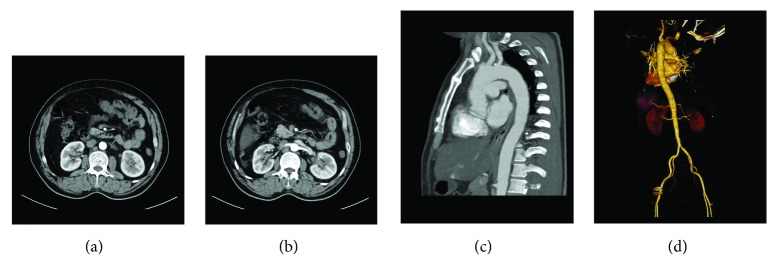
Extrapituitary involvements. (a, b) The thickening of the renal pelvis of both kidneys, with slight enhancement, is demonstrated. (c, d) Multiple annular thickening of the aorta and its primary branches is shown.

The principal clinical characteristics, endocrine abnormalities, and MRI features of IgG4-RH patients are listed in Tables 1 and 2. Isolated IgG4-RH was shown in only one case. First symptoms included hypotonic polyuria (3 cases), swollen eyelids (2 cases), fatigue and anorexia (1 case), and cough (1 case). An average of 3 (ranging from 0 to 9) extrapituitary organs were involved. The total course of IgG4-RD was 79.3 ± 75.7 months. The submandibular gland, parotid gland, and lacrimal gland, as well as the lymph nodes, were the most commonly involved organs. Other organs included the kidneys, the aorta, and the lungs.  Figure 1 illustrates images of extrapituitary involvements in this study. A marked elevation of serum IgG4 levels was observed in nine out of ten patients. The serum IgG4 level was not associated with the number of systems involved or the duration of IgG4-RD.

A pituitary biopsy was performed in patient 10. The pathological analysis revealed an inflammatory pseudotumor with lymphocyte infiltration (IgG positive and IgG4 negative). In the literature, 28 IgG4-RH cases with pituitary biopsies (including this study) were reported. Among them, 4 (14.3%) [8–10] turned out to be lacking abundant IgG4-positive cells.

As shown in Table 2, 9/10 of the IgG4-RH patients had abnormal pituitary functions. Detailed anterior and posterior pituitary function evaluation of ten patients before treatment is shown in Table 3. Five patients had panhypopituitarism, three had only posterior hypopituitarism, one had only anterior hypopituitarism, and one had normal pituitary function. In total, central diabetes insipidus was found in eight patients (80%), secondary gonadal hormone deficiency was observed in five patients (50%), secondary hypoadrenalism was observed in three patients (30%), secondary hypothyroidism was observed in two patients (20%), and growth hormone deficiency was observed in one patient (10%). The onset of anterior hypopituitarism and/or CDI occurred at the age of 46.1 ± 13.1 years, and the time course of pituitary dysfunction was 27.2 ± 31.9 months. A headache was observed in one patient, and visual defects were found in two patients, all of whom had a pituitary mass on MRI.

**Figure 2:**
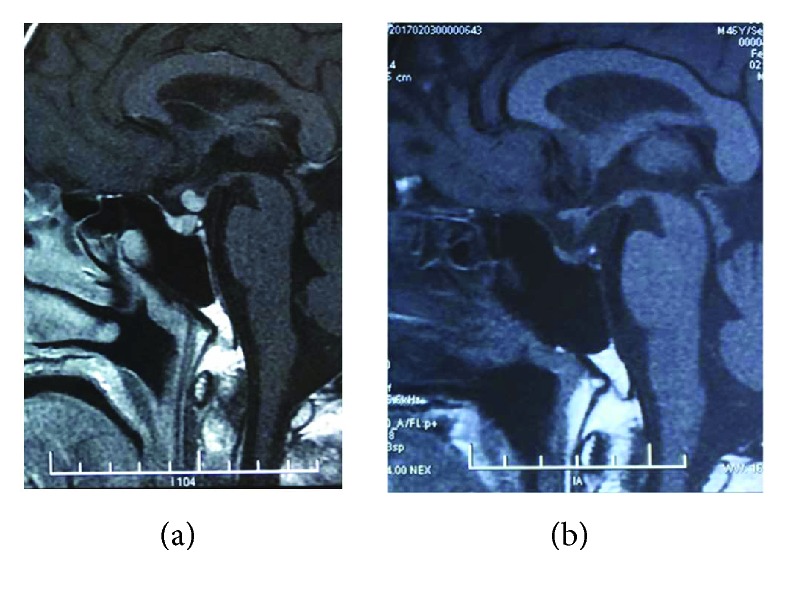
Pituitary MRI of patient 5 (a) before and (b) 1.2 months after treatment with glucocorticoids. The thickened pituitary stalk shrunk significantly.

MRI study showed one case with solitary pituitary mass and seven cases with pituitary stalk thickening. Two cases demonstrated both pituitary mass and stalk thickening. The pituitary MRIs of patient 5 at diagnosis and during follow-up are shown in Figure 2, as an illustration.

### 3.2. Treatment Outcomes

Patients 1 to 8 were followed up (Table 4). Among them, five patients were initially treated with glucocorticoids alone, while the others were treated with glucocorticoids combined with the immunosuppressive agent cyclophosphamide (CTX). All eight patients showed prompt radiological improvements and relief of symptoms in response to the initial treatment. It took an average of 1.9 months for the patients to achieve a relief of symptoms and/or shrinkage of the thickened stalk/pituitary mass. Only patients 3, 4, and 5 achieved normalized serum IgG4 levels (at 33.6, 7.7 and 2.8 months after the initial treatment started, resp.). Patient 8 showed a partial recovery of posterior pituitary function; however, the other patients did not restore pituitary function. Anterior pituitary function showed no recovery and patients followed long-term regular replacement therapy.

Two of the patients relapsed after the initial therapy. Patient 1 had a recurring headache and visual defect 3 months after he discontinued GC (lasting 2 months) by himself. He then received GC pulse therapy (methylprednisolone 500 mg i.v. for three days) followed by full-dose GC (prednisone 60 mg qd p.o.) combined with CTX (400 mg qw i.v.). His symptoms were well controlled for two months until he began GC tapering. A second immunosuppressant, tacrolimus, was then added. Patient 8 had a worsened DI 4 years after she finished 5 months of initial therapy (prednisone 50 mg qd combined with CTX 50 mg qd p.o.). She then restarted the same therapeutic regimen. Her symptoms were ameliorated, but IgG4 level raised drastically during GC tapering.

## 4. Discussion

IgG4-RH is a rare disease characterized by the presence of numerous immunohistochemically IgG4-positive plasma cells, elevated serum IgG4 levels, and thickening of the pituitary stalk [11]. In this study, we reported ten new cases of IgG4-RH, which were diagnosed based on Leporati's criteria. Applying the comprehensive diagnosis criteria of IgG4-RD [12], patients 1–8 were diagnosed as *definitive*. For patients 2, 4, 5, 6, 7, and 8, criteria 2 and 3 were fulfilled to make the diagnosis. For patients 1 and 3, the diagnosis was made according to criteria 2, 4, and 5. For patients 9 and 10, pathological analysis did not show IgG4+/IgG+ cells > 40% or IgG4+ cells > 10/HPF; therefore, they were classified as *possible* IgG4-RD. Biopsy of the pituitary lesion was only acquired in patient 10, and considering that the surgical approaches to lacrimal and salivary glands are less invasive, a biopsy of the lacrimal/submandibular mass is the best choice for making diagnosis.

In this study, patient 1 was the only case with isolated hypophysitis. There was an average of 3 extrapituitary systems involved, with the salivary glands and lymph nodes being the most common. Among patients with extrapituitary involvement, only two started with pituitary dysfunction, whereas the others had suffered from the defects of other systems for several years before CDI and/or anterior hypopituitarism occurred. Patients tend to make their first medical consultation to different departments. This emphasized the importance for physicians from various departments, ranging from stomatologists, endocrinologists, and rheumatologists to neurosurgeons, to be aware of the clinical course of IgG4-RD and the possible pituitary involvement. Endocrinologists and neurosurgeons should perform thorough physical examinations and ask for a detailed history, especially for information regarding the eyes and the salivary glands, when facing a patient with pituitary occupation and hypopituitarism. Physicians from other departments should remember the possible pituitary involvement when dealing with patients who are suspected of IgG4-RD.

Our study supports the male-dominant feature of IgG4-RH (male to female ratio of 4 : 1) and is consistent with previous reports. The age of onset was much lower in this study (46.1 ± 13.1 years) than that in the previous study (64.2 ± 13.9 years) from Japan [13]. The youngest patient diagnosed in our center was 16 years of age. A younger age of diagnosis in our center indicates a more comprehensive knowledge regarding IgG4-RH compared to that of the past few years. It also reminds us that physicians and surgeons must not neglect the possibility of IgG4-RH when dealing with younger patients including adolescents.

The presence of Mikulicz's disease (70%) and lymph node swelling (50%) is more prominent in this study compared to that in the previous review (Table 5) [13]. Headache and visual defects were not common manifestations in IgG4-RH, possibly due to the more chronic course of IgG4-RH. As for pituitary function, antidiuretic hormone deficiency (80%) was the most frequent endocrine symptom, followed by FSH/LH (50%), ACTH (30%), TSH (20%), and GH/IGF1 (10%) axis deficiency in sequence. The result was identical in Shikuma et al.'s study regarding IgG4-RH [13] last year, although the prevalence of dysfunction of the latter three axes was higher. The sequence of anterior pituitary deficiencies in IgG4-RH patients is slightly different from primary lymphocytic hypophysitis patients in a recently published Chinese study (FSH/LH > TSH > ACTH > IGF-1 axis deficiency) [14].

The histopathological analysis of the pituitary mass can be atypical. The pituitary biopsy of patient 10 revealed hypophysitis showing features of an inflammatory pseudotumor (IPT) with positive focal IgG but negative IgG4. IPT is a typical histopathological feature of IgG4-RD and is commonly shared by multiple organs involved [15]. Intracranial IgG4-related inflammatory pseudotumors were previously reported, resembling multiple meningiomas [16]. In the first histopathologically confirmed IgG4-RH case [17], the resected pituitary tumor was proven to be IPT with abundant IgG4-positive lymphoplasmacytic infiltration. An earlier report of extensive IPT of the pituitary [18] showed clusters of lymphocytes and plasma cells in histological examination, and the lesion responded promptly to glucocorticoid administration. The features discussed above are typical of IgG4-RD. However, since IgG4-RH was not well recognized until recent years, determining the serum levels of IgG4 and applying IgG4 staining were not performed in that case. Despite a negative IgG4 stain of the specimen, the typical manifestations of patient 10 in this study, including proptosis, salivary involvement, and elevated serum IgG4 level, led to the final diagnosis of IgG4-RD. This is not the first case reporting the absence of IgG4-bearing plasma cell infiltration in IgG4-RH. Of 28 IgG4-RH cases reported with pituitary biopsies (including patient 10 in the present study) [6, 8–10, 17, 19–34], four (14.3%) turned out to be lacking abundant IgG4-positive cells [8–10], while their clinical manifestations supported the diagnosis. Previous use of GC for treating other organs involved may have masked the typical plasma cell infiltration. In addition, as the accurate pathogenesis process of IgG4-RD and the role of IgG4 remain elusive, it is possible that these patients were at an initial stage of IgG4-RD when IgG4 molecules had not been synthesized. Bando et al. [35] described a patient with hypophysitis whose pituitary biopsy showed a 43% IgG4(+)/IgG(+) ratio, as well as substantial infiltration of polymorphonuclear neutrophils with giant cells; thus, the final diagnosis of this patient was granulomatosis with polyangiitis. This case further demonstrates the elusive role of IgG4-positive plasma cells. Lastly, the histopathological examination relies on the quality of the specimen, which was limited during the process of pituitary tumor excision. A false-negative histopathological result is another possible issue.

GC is considered the first-line treatment of IgG4-RD, and immunosuppressive agents are also widely used [36]. However, no standard treatment regimen has been defined. In this study, five patients with a clinical course of IgG4-RH less than nine months and a whole course of IgG4-RD less than two years were initially treated with GC at a pharmacological dose alone (recommended dose: prednisone 40–60 mg qd). Four out of the five patients were well controlled without relapse. GC in combination with CTX was prescribed for patients with a longer clinical course and for the one who failed GC monotherapy in our study. For refractory cases, a second immunosuppressant should be considered. Available choice includes methotrexate and tacrolimus. Azathioprine has also been proved effective in treating IgG4-RH [20]. It took an average of 1.9 months for all of the patients to achieve a relief of symptoms and/or shrinkage of the thickened stalk/pituitary mass. However, only three of them had normalized serum IgG4 levels. IgG4 levels may not be parallel with the severity of disease. The dose and length of GC and/or immunosuppressive administration should be based on the clinical manifestations and imaging. The two relapsing cases in this study both had short courses of initial treatment that are less than six months. GC tapering is another risk factor for symptom fluctuation. Do ensure that patients are aware of the importance of receiving an adequate length of treatment. There was one case report showing that rituximab was successfully used in treating recurrent IgG4-related hypophysitis with ophthalmopathy, where GC in combination with azathioprine failed. Notably, although with GC treatment, pituitary function/diabetes insipidus did not improve considerably (as shown in Table 4). Similar results were shown in a recent study of autoimmune hypophysitis [37]. Life-long hormone replacement therapy should be expected. For patients with bulky pituitary mass and prominent visual defects, surgical excision in order to relieve occupation effect could be a better solution. There have been a few reports regarding radiotherapy which has been used successfully in some resistant ocular adnexal IgG4-RD cases [38]. Adjunctive radiotherapy could improve patients' clinical symptoms and their ability to taper systemic GC to discontinuation [39]. However, radiotherapy for pituitary involvements has not been reported yet.

Notably, this study was limited by the inherent drawbacks of retrospective analyses. There may be missed diagnosis of GH deficiency due to the lack of dynamic hormone tests. Small sample sizes due to the low incidence rate of IgG4-RH are partially resolved by the supportive literature review.

## 5. Conclusion

IgG4-RD is a broad disease, and all physicians involved have to be aware of the possibility of pituitary dysfunction. Younger patients should be expected. The histopathological feature of pituitary gland biopsy could be atypical. For patients with a longer history, the combination of GC and immunosuppressive agents is favorable. Early and adequate courses of treatment are crucial for the management of IgG4-RH. With GC and/or immunosuppressant treatment, however, pituitary function or diabetes insipidus did not improve considerably.

## Figures and Tables

**Table 1:** Clinical characteristics of ten patients with IgG4-related hypophysitis.

ID	Age/sex	Onset age of IgG4-RH (years)	Onset age of IgG4-RD (years)	First symptoms	Serum IgG4 (mg/dl)	Other systems involved	MRI		Biopsy	Histopathology	Pituitary function		Prompt response to GC
							Thickened stalk	Pituitary mass			AH	CDI	
1	64/M	62.4	62.4	DI	199	−	+	+	N/D	N/D	+	+	+
2	61/M	60.4	60.4	Fatigue, anorexia	4680	Lymph nodes; paranasal sinus	+	+	Paranasal sinus mass	IgG4+/IgG+ > 40%, IgG4+ > 50/HPF	+	+	+
3	58/M	50.1	48.6	Swollen eyelids	5410	Submandibular gland; lacrimal gland; parotid gland; retroperitoneum	+	−	N/D	N/D	+	+	+
4	51/M	48.3	48.3	DI	1980	Submandibular gland; parotid gland; retroperitoneum	+	−	Submandibular mass	IgG4+ 20/HPF	−	+	+
5	46/M	43.3	43.3	DI	327	Lymph nodes	+	−	Lymph node	IgG4+/IgG+ > 40%, IgG4+ > 100/HPF	+	+	+
6	44/M	44.2	26.4	Swollen eyelids	1910	Pancreas; lacrimal glands; thyroid gland	+	−	Pancreas	AIP	−	+	+
7	36/M	36.4	20.6	Submandibular mass	2470	Submandibular gland; lacrimal gland; parotid gland; pericardium	+	−	Lacrimal mass	IgG4+/IgG+ > 40%, IgG4+ > 50/HPF	−	−	+
8	57/F	51.5	49.4	Cough	2250	Lacrimal gland; lung; kidney	+	−	Lacrimal mass	IgG4+ > 20/HPF	−	+	+
9	16/F	15.8	15.7	Submandibular mass	43.7	Submandibular gland; parotid gland	+	−	Submandibular mass	IgG4+/IgG+ < 40%, IgG4+ 8/HPF	+	+	n/a
10	50/M	49.0	42.0	Proptosis	426	Submandibular gland; parotid gland; lung; kidney; large artery; lymph nodes; thyroid gland; paranasal sinus; extraocular muscles	−	+	Pituitary mass	Inflammatory pseudotumor, lymphocyte infiltration, IgG(+), IgG4(−)	+	−	n/a

IgG4-RH/-RD: IgG4-related hypophysitis/disease; DI: diabetes insipidus; N/D: not determined; n/a: not available; IgG+/IgG4+: IgG-/IgG4-positive plasma cell; AH: anterior hypopituitarism; CDI: central DI; GC: glucocorticoid.

**Table 2:** Pituitary function and MRI features.

	Number of patients
Pituitary function	
Anterior and posterior dysfunction	5/10
Only posterior dysfunction	3/10
Only anterior dysfunction	1/10
Normal pituitary function	1/10
MRI features	
Panhypophysitis	2/10
Infundibulo-neurohypohysitis	7/10
Anterior hypophysitis	1/10

**Table 3:** Evaluation of pituitary function before treatment started.

Patient ID	1	2	3	4	5	6	7	8	9	10
IGF1 (age-adjusted normal range) (ng/ml)	160 (75–212)	79 (75–212)	143 (81–225)	124 (87–238)	74 (94–252)	239 (101–267)	74 (109–284)	90 (81–225)	147 (226–903)	197 (94–252)
Total testosterone (male) (ng/ml)/estradiol (female) (pg/ml)	0.85	0	0	1.56	1.33	4.55	2.91	10.3	2.86	<0.1
FSH/LH (IU/l)	12.82/6.87	0.48/0.12	0.8/0	10.79/6.53	<0.2/<0.2	9.16/4.45	6.09/3.80	47.6/21/87	0.56/1.1	0.78/0.26
Free T4 (ng/dl)	1.63	0.87	0.85	2.137	0.482	1.099	1.273	1.033	Normal	0.464
TSH (*μ*IU/ml)	1.216	0.186	1.534	0.199	1.45	3.087	1.238	2.064	Normal	4.668
Morning serum cortisol (*μ*g/dl)	0.54	1.44	Normal	10.6	13.76	29.1	11.46	14.41	<1	7.98
Morning serum ACTH (pg/ml)	Normal	7	Normal	31	22.5	44	11.6	57.4	1.34	14.9
24 h urine volume (l)	4	8	4	4.5	10	3.5	Normal	5	6	Normal
Postdehydration plasma/urine osmolality (mOsm/kg H_2_O)	287/301	151/321	267/307	n/d	n/d	n/d	n/a	n/d	167/300	n/a
Desmopressin trial	+	+	+	+	+	+	n/a	+	+	n/a

**Table 4:** Follow-up of IgG4-RH patients 1–8.

ID	Initial therapeutic regimens		Length of initial treatment (months)	T1 (months)	T2 (months)	Response			Pituitary function recovery	
	GC regimens	Immunosuppressive regimens				Clinical features	MRI features	Relapse	Anterior lobe	Posterior lobe
1	Dex 1.5 mg (tid)	n/a	2.0	2.0	n/a	+	+	+	n/a	−
2	Pred 40 mg (qd)	n/a	5.4	1.3	n/a	+	+	−	−	−
3	MP 40 mg (qd)	CTX 50 mg (qod)	48.6	6.5	33.6	+	+	−	−	−
4	MP 40 mg (qd)	CTX 400 mg (qw)	26.4	1.4	7.7	+	+	−	n/a	−
5	Pred 60 mg (qd)	n/a	7.5	1.2	2.8	+	+	−	−	−
6	Pred 40 mg (qd)	n/a	4.0	1.4	n/a	+	+	−	n/a	−
7	Pred 40 mg (qd)	n/a	12.6	0.9	n/a	+	+	−	−	n/a
8	Pred 50 mg (qd)	CTX 50 mg (qd)	5.0	1.4	n/a	+	+	+	n/a	±

GC: glucocorticoids; T1: duration of the initial therapy before having a symptomatic/radiological response; T2: duration of the initial therapy before serum IgG4 was normalized; Dex: dexamethasone; Pred: prednisone; MP: methylprednisolone; CTX: cyclophosphamide; n/a: not applicable; +: remission; ±: partial; −: negative.

**Table 5:** Clinical features of this study and a previous review.

	This study	Shikuma et al. [13]
Mean age of onset (years)	46.1 ± 13.1	64.2 ± 13.9
Extrapituitary lesions		
Retroperitoneal fibrosis	20%	26.2%
Mikulicz's disease, Küttner's tumor	70%	25.0%
Lymph node swelling	50%	23.8%
Lung inflammatory pseudotumor interstitial pneumonia	20%	20.2%
Autoimmune pancreatitis	10%	14.3%
Tubulo-interstitial nephritis, kidney inflammatory pseudotumor	20%	11.9%
Hypertrophic pachymeningitis	0	8.3%
Orbital pseudotumor iridocyclitis	0	8.3%
Liver inflammatory pseudotumor	0	3.6%
Nasal sinus inflammatory pseudotumor	20%	2.4%
Sclerosing cholangitis	0	2.4%
Riedel's thyroiditis	20%	1.2%
Inflammatory aneurism	10%	1.2%
Gastric wall thickness	0	1.2%
Iliopsoas muscle	0	1.2%
Prostatitis	0	1.2%
Pituitary hormone deficiency		
ADH	80%	72.0%
FSH/LH	50%	48.8%
ACTH	30%	47.6%
TSH	20%	41.5%
GH	10%	41.5%
MRI features		
Thickened stalk alone	70%	21.4%
Pituitary mass alone	10%	14.3%
Both	20%	64.3%
