# Erratum to “Hypophyseal Involvement in Immunoglobulin G4-Related Disease: A Retrospective Study from a Single Tertiary Center”

**DOI:** 10.1155/2018/3457046

**Published:** 2018-09-06

**Authors:** Yang Liu, Linjie Wang, Wen Zhang, Hui Pan, Hongbo Yang, Kan Deng, Lin Lu, Yong Yao, Shi Chen, Xiaofeng Chai, Feng Feng, Hui You, Zimeng Jin, Huijuan Zhu

**Affiliations:** ^1^Department of Neurosurgery, Peking Union Medical College Hospital, Chinese Academy of Medical Science and Peking Union Medical College, Beijing 100730, China; ^2^Key Laboratory of Endocrinology of National Health and Family Planning Commission, Department of Endocrinology, Peking Union Medical College Hospital, Chinese Academy of Medical Science and Peking Union Medical College, Beijing 100730, China; ^3^Department of Rheumatology, Peking Union Medical College Hospital, Chinese Academy of Medical Science and Peking Union Medical College, Beijing 100730, China; ^4^Department of Radiology, Peking Union Medical College Hospital, Chinese Academy of Medical Science and Peking Union Medical College, Beijing 100730, China

In the article titled “Hypophyseal Involvement in Immunoglobulin G4-Related Disease: A Retrospective Study from a Single Tertiary Center” [1], the first, second, and third affiliations were incomplete. The corrected affiliations are shown above.
